# Ultra‐Steep‐Slope High‐Gain MoS_2_ Transistors with Atomic Threshold‐Switching Gate

**DOI:** 10.1002/advs.202104439

**Published:** 2022-01-17

**Authors:** Jun Lin, Xiaozhang Chen, Xinpei Duan, Zhiming Yu, Wencheng Niu, Mingliang Zhang, Chang Liu, Guoli Li, Yuan Liu, Xingqiang Liu, Peng Zhou, Lei Liao

**Affiliations:** ^1^ Key Laboratory for Micro/Nano Optoelectronic Devices of Ministry of Education& Hunan Provincial Key Laboratory of Low‐Dimensional Structural Physics and Devices School of Physics and Electronics Hunan University Changsha 410082 China; ^2^ State Key Laboratory of ASIC and System Department of Microelectronics Fudan University Shanghai 200433 P. R. China; ^3^ College of Semiconductors (College of Integrated Circuits) Hunan University Changsha 410082 China

**Keywords:** high gain, inverter, resistive gate, steep slope, threshold swing

## Abstract

The fundamental Boltzmann limitation dictates the ultimate limit of subthreshold swing (SS) to be 60 mV dec^−1^, which prevents the continued scaling of supply voltage. With atomically thin body, 2D semiconductors provide new possibilities for advanced low‐power electronics. Herein, ultra‐steep‐slope MoS_2_ resistive‐gate field‐effect transistors (RG‐FETs) by integrating atomic‐scale‐resistive filamentary with conventional MoS_2_ transistors, demonstrating an ultra‐low SS below 1 mV dec^−1^ at room temperature are reported. The abrupt resistance transition of the nanoscale‐resistive filamentary ensures dramatic change in gate potential, and switches the device on and off, leading to ultra‐steep SS. Simultaneously, RG‐FETs demonstrate a high on/off ratio of 2.76 × 10^7^ with superior reproducibility and reliability. With the ultra‐steep SS, the RG‐FETs can be readily employed to construct logic inverter with an ultra‐high gain ≈2000, indicating exciting potential for future low‐power electronics and monolithic integration.

## Graphical abstract

Ultra‐steep‐slope MoS_2_ resistive‐gate field‐effect transistors (RG‐FETs) by integrating atomic‐scale resistive filamentary with conventional MoS_2_ transistors, demonstrating an ultra‐low SS below 1 mV dec^−1^ at room temperature are reported. Ultra‐high gain ≈2000 is demonstrated in the fabricated inverter.

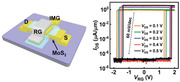

## 1 Introduction

Continuous miniaturization of traditional silicon‐based metal‐oxide‐semiconductor field‐effect transistors (MOSFETs) has facilitated aggressive high‐performance integrated circuits with ever‐increasing speed, integration density, and low power consumption. Improved MOSFETs performance requires to reduce the supply voltage (*V*_DD_) and the threshold voltage (*V*_TH_) to maintain high overdrive factor (*V*_DD_−*V*_TH_).^[^ ^1^, ^2^ ^]^ However, subthreshold swing (SS) is fundamentally limited to above 60 mV dec^−1^ at room temperature, because of Boltzmann distribution of carrier, which leads to exponential growth of leakage current and static power consumption.^[^ ^3^ ^]^ Therefore, reducing SS has become one of the critical challenges for large‐scale high‐performance integrated circuit development.^[^ ^4^ ^]^ Several steep‐slope devices based on innovative structure or transport mechanisms, such as tunnel FETs (TFETs)^[^ ^5^, ^6^ ^]^ and negative capacitance FETs (NCFETs),^[^ ^7^ ^]^ have been proposed to combat the “Boltzmann tyranny,” which have SS below 60 mV dec^−1^ at room temperature. However, in TFETs, because carriers are injected into the channel through band‐to‐band tunneling, drive current is greatly reduced.^[^ ^8^ ^]^ While in NCFETs, ferroelectric materials are prone to fatigue in the electric field, so the applications are affected by long‐term stability.^[^ ^9^ ^]^ Therefore, fabricating FETs with ultra‐high on–off ratio and ultra‐low SS is an urgent issue for low‐power circuits. Collective interactions in functional materials potentially enable augment current of state‐of‐the‐art transistors, which provides unique routes to overcome conventional limits for high‐performance devices. Insulator‐to‐metal transition threshold switch (TS) with connection with FET was previously reported, and realized abrupt resistivity switching for steep‐slope operation and demonstrates SS below 60 mV dec^−1^ at room temperature. However, due to the series connection of the memristor and transistor, high drive voltage and low on‐state output current are needed.^[^ ^10^, ^11^, ^12^ ^]^

Herein, black phosphorus (BP)‐based filamentary TS resistive gate (RG) is integrated on top of a top‐gated MoS_2_ FETs, in which atomic‐scale filament formation/dissolution (in the gate region) can be induced by a low triggering voltage. Therefore, RG‐FETs exhibit ultra‐steep slope and demonstrate SS below 1 mV dec^−1^ at room temperature. By rationally designing threshold voltage (*V*_TH_) of common MoS_2_ transistors with HfO_2_ dielectric layer, gate potential from RG can readily exceed *V*_TH_. Although the conduction current of RG‐FETs is still dominated by carrier injection over potential barrier like MOSFET, abrupt resistance transition of the TS leads to obvious change of the internal metal gate (IMG) voltage, leading to ultra‐low SS. Therefore, it could overcome fundamental thermionic limitation and maintain competitive on‐current with that of MoS_2_ MOSFETs.

## 2 Results and Discussion

### 2.1 RG‐FETs Fabrication

**Figure 1:**
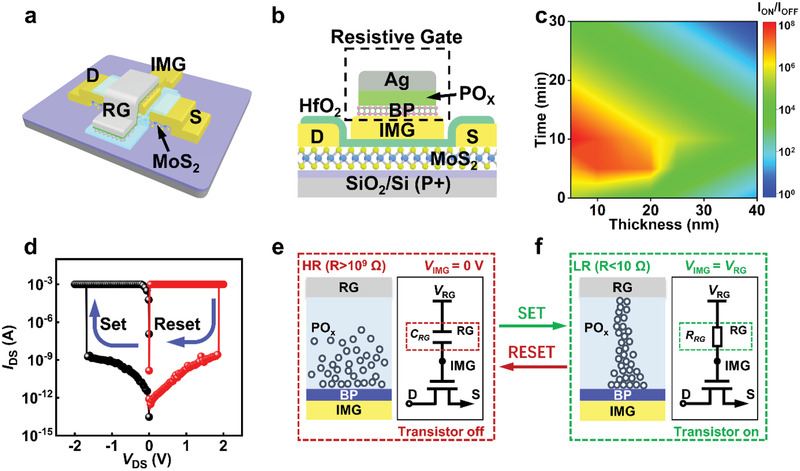
Schematic image and mechanism illustration of the ultra‐steep‐slope MoS_2_ RG‐FETs. a) Schematic image of the MoS_2_ RG‐FETs. b) Cross‐sectional illustration of MoS_2_ RG‐FETs. c) 2D plot of the relationship between TS versus ozone treatment time (*Y*‐axis) and the thickness of BP (*X*‐axis) that influence on/off ratio of MoS_2_ RG‐FETs. d) Typical *I*–*V* characteristics of BP memristor. e) Operation principle of the RG‐FETs.

**Figure** **1**a schematically presents the device structure of MoS_2_ RG‐FETs. Mechanical‐exfoliated MoS_2_ flakes with thickness of three to five layers are selected as the channel to ensure high carrier mobility and low contact resistance.^[^ ^13^, ^14^ ^]^ Figure 1b exhibits the cross‐sectional view of MoS_2_ RG‐FETs, and fabrication processes are detailed in the Supporting Information (Figure S1, Supporting Information). The BP/PO*_x_* TS is formed by oxidation of 10 nm BP flake via ozone treatment.^[^ ^15^ ^]^ Large on–off ratio and low transition voltage of TS are essential for abruptly changing IMG potential. Figure 1c illustrates the dependence of ozone treatment time and BP thickness on the on/off ratio of the TS, and 10 nm BP with 10 min ozone treatment is selected as the TS layer. The resistive characteristics of BP TS are shown in Figure 1d. And the retention and endurance characteristics are present in the Supporting Information (Figure S2, Supporting Information), indicating high reliability and reproducibility. To illustrate the formation principle of two current states, Figure 1e plots formation/rupture of conductive filament and corresponding equivalent circuit diagram. Briefly, at low external voltage below the transition voltage of BP memristor, there is no current path formed in the BP oxide or the TS has been ruptured. The memristor is in high resistance state (HRS) and presents a high resistance over 10^9^ Ω, and thus the IMG is insulated from the external gate. Therefore, the transistor is in off‐state. When a high voltage above the transition voltage of BP‐based memristor, the formation of TS makes the BP oxide to have a low resistance below 10 Ω at low resistance state (LRS). At this time, the IMG potential is nearly equivalent to the external gate voltage, and thus the transistor is on, as shown in Figure 1f.

### 2.2 Mechanism and Electrical Performance of the RG‐FETs

**Figure 2:**
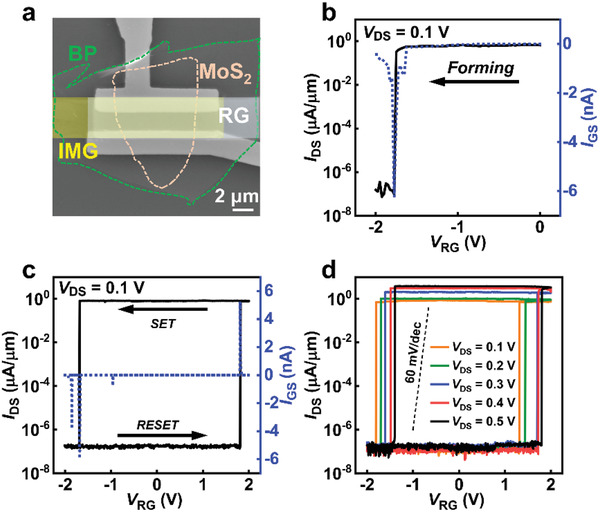
Electrical performance of the MoS_2_ RG‐FETs. a) Scanning electron microscope (SEM) image of the MoS_2_ RG‐FETs, and the scale bar is 2 µm. b) The transfer characteristic of the MoS_2_ RG‐FETs during the forming process of TS. The blue dash dots present the gate current of the transistor. c) The transfer characteristic of the MoS_2_ RG‐FETs as the TS transits from “*SET*” to “*RESET*,” and the blue dash dots are the gate current of the transistor. d) The transfer characteristic of the MoS_2_ RG‐FETs with different source–drain voltage (*V*_DS_).

Typical scanning electron microscope (SEM) image of MoS_2_ RG‐FETs is shown in **Figure** **2**a. The resistive switching effect of the TS is caused by redox reactions, which leads to the formation and rupture of a few nanometers thin conductive filament connecting RG and IMG electrodes, and the oxygen vacancies play a critical role. As shown in Figure 2b, the source–drain bias (*V*_DS_) is kept at 0.1 V and the voltage applied on RG (*V*_RG_) sweeps from 0 to −2.0 V. At beginning, the initial channel current (*I*_DS_) is 1 µA µm^−1^ when *V*_RG_ = 0 V and sharply decreases to 10^–7^ µA µm^−1^ when *V*_RG_ = −1.75 V. At the same time, the gate current (*I*_GS_) has the same trend as *I*_DS_, which proves that ultra‐steep slope is achieved by conductive filament formation and rupture, as shown in Figure 2b. Forward and backward sweep is applied on device in the range between −2 and 2 V, as shown in Figure 2c. When resistance state of the TS transforms from LRS to HRS, corresponding to the “*RESET*” process of the TS, *I*_DS_ is rapidly drawn back to high level. When resistance state of the TS transforms from HRS back to LRS, corresponding to the “*SET*” process of the TS, *I*_DS_ abruptly drops down to low level. That is because oxygen vacancies accumulation induces conductive filament formation, and the IMG voltage approaches to the external RG voltage. By using ultra‐thin HfO_2_ as dielectric layer, the *V*_TH_ of the MoS_2_ FETs is −0.7 V (Figure S3, Supporting Information), which can afford IMG voltage suddenly transitions between high and low voltage level. Figure 2d shows the transfer characteristics of MoS_2_ RG‐FETs with different *V*_DS_. Both *V*_TH_ and on/off ratio increase with *V*_DS_, which is induced by disproportionate increment of the IMG voltage from the external RG.

### 2.3 Statistical Analysis of MoS_2_ RG‐FET and Performance Comparison

**Figure 3:**
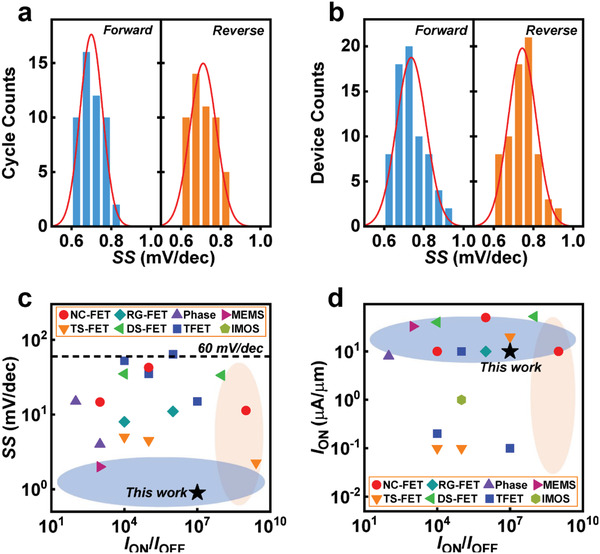
Statistical data of the MoS_2_ RG‐FETs. a) The cycle‐to‐cycle variations of forward sweeping SS and reverse sweeping SS at *V*_DS_ = 0.1 V. b) The device‐to‐device deviations of forward sweeping SS and reverse sweeping SS at *V*_DS_ = 0.1 V. c,d) SS and *I*_ON_ comparisons of various steep‐slope FETs, respectively, including TFETs,^[^ ^16^, ^17^, ^18^, ^19^, ^20^ ^]^ impact‐ionization FETs (IMOS‐FETs),^[^ ^21^ ^]^ Dirac‐source FETs (DS‐FET),^[^ ^8^, ^9^ ^]^ phase‐transition FET (phase‐FETs),^[^ ^22^, ^23^ ^]^ resistive‐switching FETs (TS‐FETs),^[^ ^10^, ^24^, ^25^ ^]^ NC‐FETs,^[^ ^26^, ^27^, ^28^ ^]^ nano‐electro‐mechanical FETs (NEM‐FETs),^[^ ^29^ ^]^ RG‐FETs,^[^ ^11^, ^12^ ^]^ and this work.

Statistical analysis can illustrate the reliability and reproducibility of MoS_2_ RG‐FETs. The statistical analysis for the forward and reverse SS is conducted in cycle‐to‐cycle (50 cycles) and device‐to‐device (70 devices) at *V*_DS_ = 0.1 V, as shown in **Figure** **3**a,b, respectively. The extracted SS is concentrated on a very small region, from 0.6 to 1.0 mV dec^−1^, which means that abrupt switching behavior of RG‐FETs has minimized cycle‐to‐cycle variations and device‐to‐device deviations, indicating high stability and uniformity. The Gaussian distributions of forward and reverse SS in Figure 3a,b show the average SS values of 0.7 and 0.71 mV dec^−1^ in cycle‐to‐cycle test and 0.73 and 0.74 mV dec^−1^ in device‐to‐device deviation. Figure S4a in the Supporting Information shows cycle‐to‐cycle variation of channel current with consecutive “*RESET*” operations. Figure S4b in the Supporting Information is the dependence of *I*_ON_ (*y*‐axis) and *V*_TH_ (*x*‐axis) on *V*_DS_. Figure S4c in the Supporting Information shows relationship between *V*_RG_ and on/off ratio. All of these indicate the RG‐FETs obtain robust stability and reliability. The performance comparison among the steep‐slope transistors is shown in Figure 3c,d. Compared with other classical low SS prototype devices, including TFETs,^[^ ^16^, ^17^, ^18^, ^19^, ^20^ ^]^ impact‐ionization FETs (IMOS‐FETs),^[^ ^21^ ^]^ Dirac‐source FETs (DS‐FET),^[^ ^8^, ^9^ ^]^ phase‐transition FET (phase‐FETs),^[^ ^22^, ^23^ ^]^ resistive‐switching FETs (TS‐FETs),^[^ ^10^, ^24^, ^25^ ^]^ NC‐FETs,^[^ ^26^, ^27^, ^28^ ^]^ nano‐electro‐mechanical FETs (NEM‐FETs),^[^ ^29^ ^]^ RG‐FETs,^[^ ^11^, ^12^ ^]^ this work presents extremely steep SS below 1 mV dec^−1^ and high switching ratio of 2.76 × 10^7^, indicating MoS_2_ RG‐FETs are promising for high‐performance low‐power electronics.

### 2.4 Ultra‐High Gain Inverter

**Figure 4:**
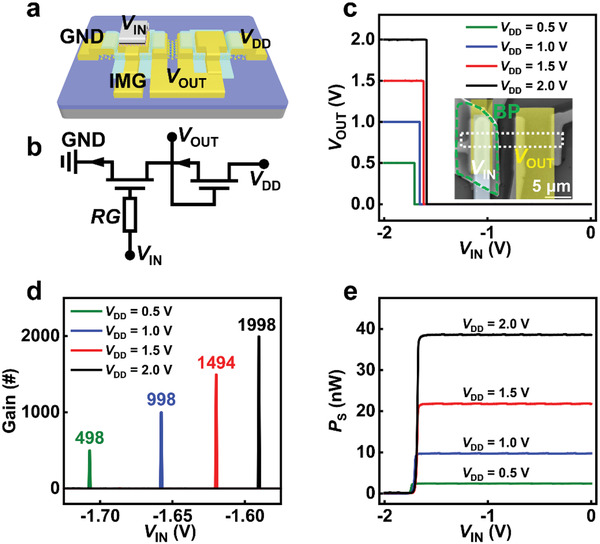
Ultra‐high gain inverter based on MoS_2_ RG‐FETs. a) Schematic image of the inverter. b) Circuit diagram of the inverter based on MoS_2_ RG‐FETs. c) Electrical performance of the inverter at different applied voltage (*V*_DD_). The inset is the corresponding SEM image. d) Voltage gain of the inverter at different *V*_DD_. e) Dynamic power consumption (*P*_d_) as a function of the input voltage *V*_IN_ under different *V*_DD_.

Direct‐coupled FET logic technology is alternative architecture for constructing low‐power circuit.^[^ ^30^ ^]^ Based on the ultra‐high and super‐steep on/off ratio, an ultra‐high gain inverter is fabricated. As shown in **Figure** **4**a, MoS_2_ RG‐FET with channel length of 0.5 µm serves as the switch of inverter, and the load is realized by common MoS_2_ FETs working at saturation state. This inverter is completed by connecting the load transistor (connected to *V*_DD_) and switch transistor in series. The connection between switch transistor and load transistor gate acts as the output terminal, as shown in Figure 4b. Figure 4c shows electrical performance of the inverter at different *V*_DD_, the input voltage (*V*_IN_) sweeps from 0 to −2 V (100 step s^−1^ with a voltage span of 0.5 mV per step), and the inset is corresponding SEM image. With ultra‐steep‐slope characteristic of MoS_2_ RG‐FETs, abruptly turn on of the switch MoS_2_ RG‐FETs leads to ultra‐sharp transition between high and low level of the inverter. Figure 4d indicates voltage gain of the inverter, and an ultra‐high gain of 1998 at *V*_DD_ = 2.0 V is obtained, representing a highest value among the previously reported 2D materials‐based inverters.^[^ ^2^ ^]^ Besides high voltage gain, Figure 4d shows that the inverter has ultra‐low dynamic power consumption (*P*_S_ = *V*_DD_ × *I*_DD_) and its power consumption is less than 40 nW at *V*_DD_ = 2.0 V. The static power consumption is calculated by *P*_s_ = *V*_s_^2^/(*R*_L_ +*R*_on_),^[^ ^31^ ^]^ where *R*_L_ and *R*_on_ are load resistance and resistance of RG‐FETs, respectively. At high input level, the static power consumption is equal to *P*_s_ = *V*_s_^2^/*R*_L_, so its maximum static power consumption is about 0.2 nW at *V*_DD_ = 2.0 V. While at low input level, there is only leakage current in the inverter, so the static power consumption is equal to 0 W. Moreover, by thinning the dielectric layer thickness and reducing the transition voltage of TS, the hysteresis and *V*_TH_ of the RG‐FETs can be further optimized. All of these indicate the fabricated RG‐FETs are promising for high‐performance low‐power electronics.

## 3 Conclusions

In summary, ultra‐steep‐slope MoS_2_ RG‐FETs are constructed by introducing atomic resistive gate that is connected with the IMG. The devices exhibit an ultra‐low SS value below 1 mV dec^−1^ at room temperature, indicating superior reliability and reproducibility. Based on abruptly switching characteristic of MoS_2_ RG‐FETs, high‐performance inverters with 1998 voltage gain are obtained. Because the proposed strategy still relies on carrier injection mechanism, fabrication processes are compatible with traditional planner processes. Therefore, MoS_2_ RG‐FETs show the potential of promising candidate for future monolithic integration and advanced low‐power electronics.

## 4 Experimental Section

### Device Fabrication

5 nm thick MoS_2_ nanoflakes were exfoliated mechanically with Scotch tape and transferred on silicon (Si) substrates with a thermally grown 300 nm thick SiO_2_ layer. And copolymer was spin‐coated at a speed of 3000 rpm and was baked on a hot plate at 150 °C for 1 min, and then polymethyl methacrylate 495k was spin‐coated at 3000 rpm and baked at 150 °C for 5 min. Subsequently, the source and drain contact regions of MoS_2_ RG‐FETs were defined by a standard electron‐beam lithography (EBL), followed by thermal evaporation of Cr/Au (10/30 nm), and lift‐off process. Then, 5 nm thick dielectric HfO_2_ layer was prepared via atomic layer deposition. The IMG electrodes of the MoS_2_ FETs were fabricated by a second EBL process, metallization of Au (30 nm), and lift‐off process. After that, 10 nm thick BP was transferred onto the IMG electrode by a physical dry transfer process, followed by 5 min ozone treatment to form an ultra‐thin resistive oxide onto the surface. Finally, the resistive gate electrodes of MoS_2_ RG‐FETs were defined at the desired position by a third EBL process, metallization of Ag (100 nm), and lift‐off process.

### Materials Characterization and Electrical Measurements

SEM images were conducted on JOEL IT300 operated at 20 kV, and the EBL was carried out on a Raith pattern generator SEM combination. Electrical measurements of the transistors were performed on a probe station equipped with Agilent B1500A semiconductor parameter analyzer under a vacuum environment.

## Conflict of Interest

The authors declare no conflict of interest.

## Supporting information

Supporting information

## Data Availability

Research data are not shared.
