# Supplementary material for: Ultra‐Steep‐Slope High‐Gain MoS2 Transistors with Atomic Threshold‐Switching Gate
Source: Adv Sci (Weinh). 2022 Jan 17;9(8):2104439. doi: 10.1002/advs.202104439 (PMC8922111; doi:10.1002/advs.202104439)
Supplement: Supplementary file 1 — Supporting information [file ADVS-9-2104439-s001.pdf]

ADVANCED SCIENCE
Open Access

Supporting Information

for *Adv. Sci.*, DOI 10.1002/advs.202104439

Ultra-Steep-Slope High-Gain MoS_2 Transistors with Atomic Threshold-Switching Gate

Jun Lin, Xiaozhang Chen, Xinpei Duan, Zhiming Yu, Wencheng Niu, Mingliang Zhang, Chang Liu, Guoli Li, Yuan Liu, Xingqiang Liu, Peng Zhou* and Lei Liao**

ADVANCED SCIENCE
Open Access

Supporting Information

for *Adv. Sci.*, DOI: 10.1002/advs.202104439

Ultra-Steep-Slope High-Gain MoS_2 Transistors with Atomic Threshold-Switching Gate

Jun Lin, Xiaozhang Chen, Xinpei Duan, Zhiming Yu, Wencheng Niu, Mingliang Zhang, Chang Liu, Guoli Li, Yuan Liu, Xingqiang Liu, Peng Zhou,* Lei Liao**

Supporting Information

Ultra-Steep-Slope High-Gain MoS_2 Transistors with Atomic Threshold-Switching Gate

Jun Lin, Xiaozhang Chen, Xinpei Duan, Zhiming Yu, Wencheng Niu, Mingliang Zhang, Chang Liu, Guoli Li, Yuan Liu, Xingqiang Liu, * *Peng Zhou,* * *Lei Liao* *

Figure S1. The fabrication processes of the RG-FETs.

Figure S2. Retention and endurance characteristics of the BP memristors.

Figure S3. Performance of the MoS_2 FETs with 5 nm HfO_2 as dielectric layer.

Figure S4. Statistical data of the MoS_2 RG-FETs.

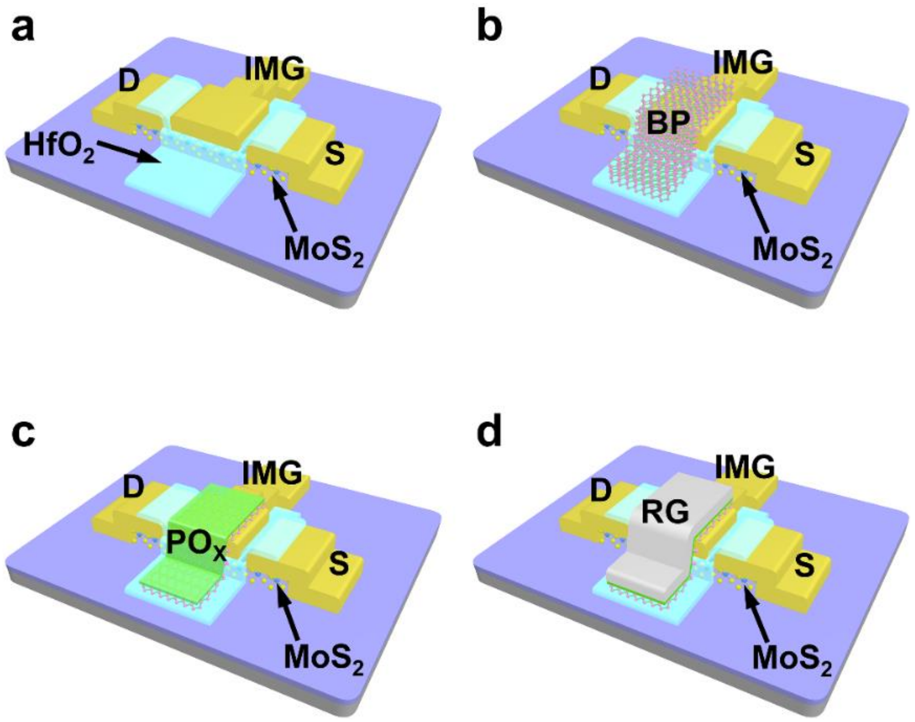

Figure S1. The fabrication processes of the RG-FETs. (a) Schematic image of the top-gate MoS_2 FETs on silicon (Si) substrates with 300 nm-thick SiO_2 layer. (b) 10 nm-thick black phosphorus is transferred on the IMG electrode via a physical dry transfer approach. (c) Ozone treatment is introduced to form phosphorus oxide. (d) Schematic image of the fabricated MoS_2 RG-FETs.

5 nm-thick MoS_2 nanoflakes were exfoliated mechanically with Scotch tape and transferred on silicon (Si) substrates with 300 nm-thick SiO_2 layer. And copolymer is spin-coated at a speed of 3000 rpm and was baked on a hot plate at 150 °C for 1 minutes, and then Polymethyl methacrylate (PMMA) 495k was spin-coated at 3000 rpm and baked at 150 °C for 5 minutes. Subsequently, the source and drain contact regions of MoS_2 RG-FETs were defined by a standard electron-beam lithography, followed by thermal evaporation of Cr/Au (10/30 nm), and lift-off process. Then, 5nm-thick dielectric HfO_2 layer was prepared via atomic layer deposition (ALD). The IMG electrodes of the MoS_2 FETs were fabricated by a second EBL process, metallization of Au (30 nm) and lift-off process, as shown in Figure S1 a. After that, as shown in Figure S1b, 10 nm-thick black phosphorus is transferred onto the IMG electrode by a physical dry transfer process. And then 5 minutes ozone treatment is introduced to form an ultra-thin resistive oxide onto the surface, as shown in Figure S1c. Finally, the resistive gate electrodes of MoS_2 RG-FETs were defined at the desired position by an EBL process, metallization of Ag (100nm) and lift-off processes, as shown in Figure S1d.

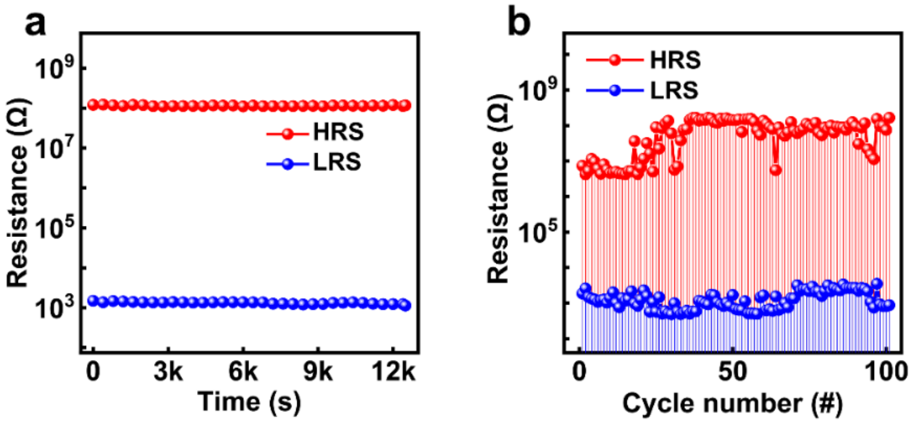

Figure S2. Retention and endurance characteristics of the BP memristors. (a) Time retention test result of the memristor up to 12000 s, and the read voltage is set to be 0.12 V. (b) Endurance characteristics of the BP memristors.

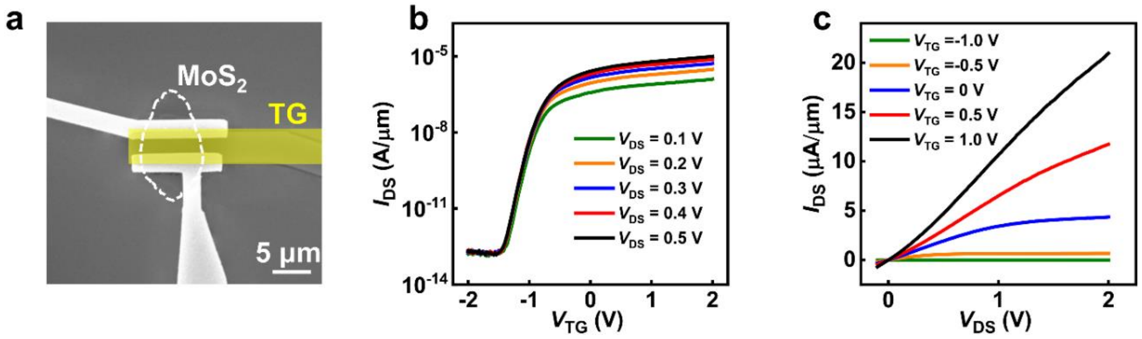

Figure S3. Performance of the MoS_2 FETs with 5 nm HfO_2 as dielectric layer. (a) SEM image of MoS_2 FETs, which is performed on JOEL IT300 operated at 20 kV, and the EBL is carried out on a Raith pattern generator-SEM combination. (b) Transfer characteristic plot and output characteristic curve (c) are performed on a probe station equipped with Agilent B1500A semiconductor parameter analyzer under a vacuum environment.

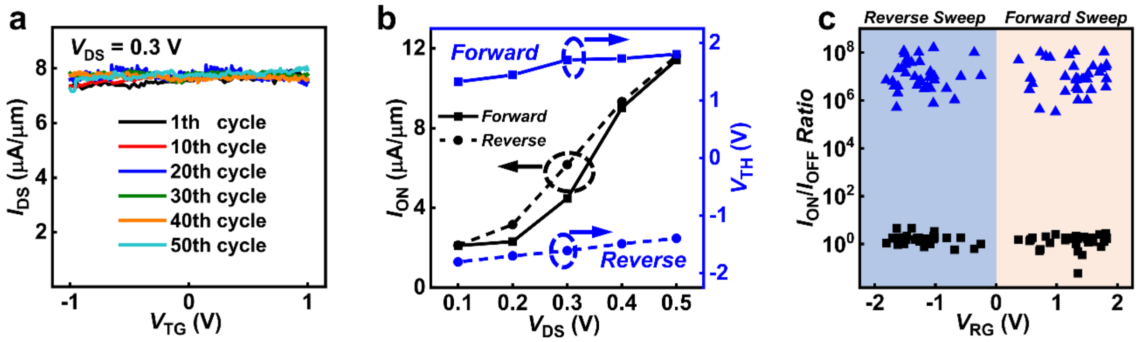

Figure S4. Statistical data of the MoS_2 RG-FETs. (a) Cycle-to-cycle variation of channel current with consecutive "*RESET*" operations. (b) The plots of I_{ON} and V_{TH} of the RG-FETs at different V_{DS} values. (c) The on-off ratio distribution versus V_{RG} of the MoS_2 RG-FETs.
